# Diversity, distribution and different habitat use among the tropical freshwater eels of genus *Anguilla*

**DOI:** 10.1038/s41598-017-07837-x

**Published:** 2017-08-08

**Authors:** Takaomi Arai, Siti Raudah Abdul Kadir

**Affiliations:** 1 Environmental and Life Sciences Programme, Faculty of Science, Universiti Brunei Darussalam, Jalan Tungku Link, Gadong BE 1410 Brunei Darussalam; 2 Institute of Oceanography and Environment, Universiti Malaysia Terengganu, 21030 Kuala Terengganu, Terengganu Malaysia

## Abstract

Along with the mysteries of their ecology, freshwater eels have fascinated biologists for centuries. However, information concerning species diversity, geographic distribution, and life histories of the tropical anguillid eels in the Indo-Pacific region are highly limited. Comprehensive research on the species composition, distribution and habitat use among tropical anguillid eels in the Peninsular Malaysia were conducted for four years. A total of 463 specimens were collected in the northwestern peninsular area. The dominant species was *A. bicolor bicolor* constituting of 88.1% of the total eels, the second one was *A. bengalensis bengalensis* at 11.7%, while *A. marmorata* was the least abundant at 0.2%. *A. bicolor bicolor* was widely distributed from upstream to downstream areas of the rivers. In comparison, *A. bengalensis bengalensis* preferred to reside from the upstream to midstream areas with no tidal zones, cooler water temperatures and higher elevation areas. The habitat preference might be different between sites due to inter-species interactions and intra-specific plasticity to local environmental conditions. These results suggest that habitat use in the tropical anguillid eels might be more influenced by ambient environmental factors, such as salinity, temperature, elevation, river size and carrying capacity, than ecological competition, such as interspecific competition.

## Introduction

Nineteen species/subspecies of freshwater eels have been reported worldwide, 13 of which inhabit tropical regions^1, 2^ that are globally distributed in temperate, tropical, and subtropical areas. In tropical areas, seven species occur in the Western Pacific region around Indonesia and Malaysia^1, 3, 4^. The life cycle of the freshwater eel has five principal stages: leptocephalus, glass eel, elver, yellow eel and silver eel. The larvae, leptocephali, drift on ocean currents and are transported by the current. The leptocephali leave the ocean currents after metamorphosing into glass eels, and then typically migrate upstream as elvers four to eight months after hatching^5^ to grow in fresh water habitats during the yellow eel stage (immature stage). After the upstream migration, the elvers become yellow eels and live in fresh water habitats such as rivers and lakes. However, because individuals of several anguillid species have been found to remain in estuarine or marine habitats, it appears that not all anguillid eels enter freshwater environments; these species display more of an apparent facultative catadromy^6, 7^. Then, during the silver eel stage (early maturing stage) in autumn and winter, their gonads begin to mature, and they start their downstream migration back to the spawning area in the ocean where they eventually spawn and die.

In general, freshwater eels are divided into temperate and tropical eels based on their distribution range^2^. Molecular phylogenetic studies on freshwater eels have revealed that tropical eels are the most basal species originating in the Indonesian and Malaysian regions and that freshwater eels radiated out from the tropics to colonize the temperate regions^8^. This suggests that tropical freshwater eels are more closely related to the ancestral form than their temperate counterparts. Thus, studying the biological aspects of tropical eels, may provide clues for understanding the life history and nature of primitive forms of freshwater eels, and how the worldwide distribution of the anguillid eels became established.

The drastic decline in recruitment of temperate anguillid eel species (*Anguilla anguilla* in Europe; *A. rostrata* in North America; *A*. *japonica* in East Asia) in recent times has caused serious problems for maintaining sustainable levels of adult organisms^9–11^. Tropical eels are becoming the major target species for satisfying the high demand for eel products. Growing concern for many of the tropical anguillid species, particularly those inhabiting parts of the Western Central Pacific, was reflected in the assessments of species within this area^12^. However, very little is known about the species diversity, geographic distribution, and life histories of the many tropical eel species that are found in the Indo-Pacific region. A systematic, phylogenetic and geographical study by Ege^1^ has revealed that the highest diversity of Anguillidae occurs in the Indonesian and Malaysian waters. Recently, several studies have investigated spawning ecology^13, 14^, early life history^4, 5, 15–17^ and migration^18, 19^ of tropical eels in Indonesia. The findings from these studies suggest that the characteristics of both life history and ecology differ between temperate and tropical eels. The year-round spawning of the tropical species and the corresponding constant larval growth extend the period of recruitment to estuarine habitats to the whole year^4, 5, 14, 17, 20^. Such spawning ecology and recruitment mechanisms in tropical eels are enabled by local short-distant migrations^13^. For temperate eels, however, the retention of their spawning areas in the tropics requires them to migrate thousands of kilometres^21^ to have distinct seasonal patterns of downstream migration, spawning in the open ocean, and recruitment of glass eels.

There is relatively little information available on various aspects of the eel biology including species composition, geographic distribution, life history and migration in tropical waters. Among the tropical region, Malaysia is one of the important geographical niches of Anguillidae. Thus research on eel biology in Malaysia could provide details about their species diversity, evolutionary pathways, and life history. According to past studies, tropical eel species *Anguilla bicolor bicolor, A. marmorata* and *A. bengalensis bengalensis* have been found in Peninsular Malaysia^22–30^. However, these studies have mainly focused on fish records, re-examination and identification of the anguillid eels. Little effort has been made to examine the species diversity, geographic distribution, habitat ecology and life history of the anguillid eels in Malaysia and Indo-Pacific region.

In the present study, comprehensive research on species diversity and geographic distribution of the anguillid eel species of Peninsular Malaysia has been done by employing accurate methods using molecular genetic analysis after conducting morphological observations. Furthermore, habitat use, segregation and preference by the tropical anguillid eels were also examined by considering environmental factors such as salinity, water temperature, tidal effects and elevation of the habitats. This study also discusses the mechanism of habitat use and segregation by sympatric species of tropical anguillid eels inhabiting a river system.

## Results

### Species composition in Peninsular Malaysia

**Figure 1:**
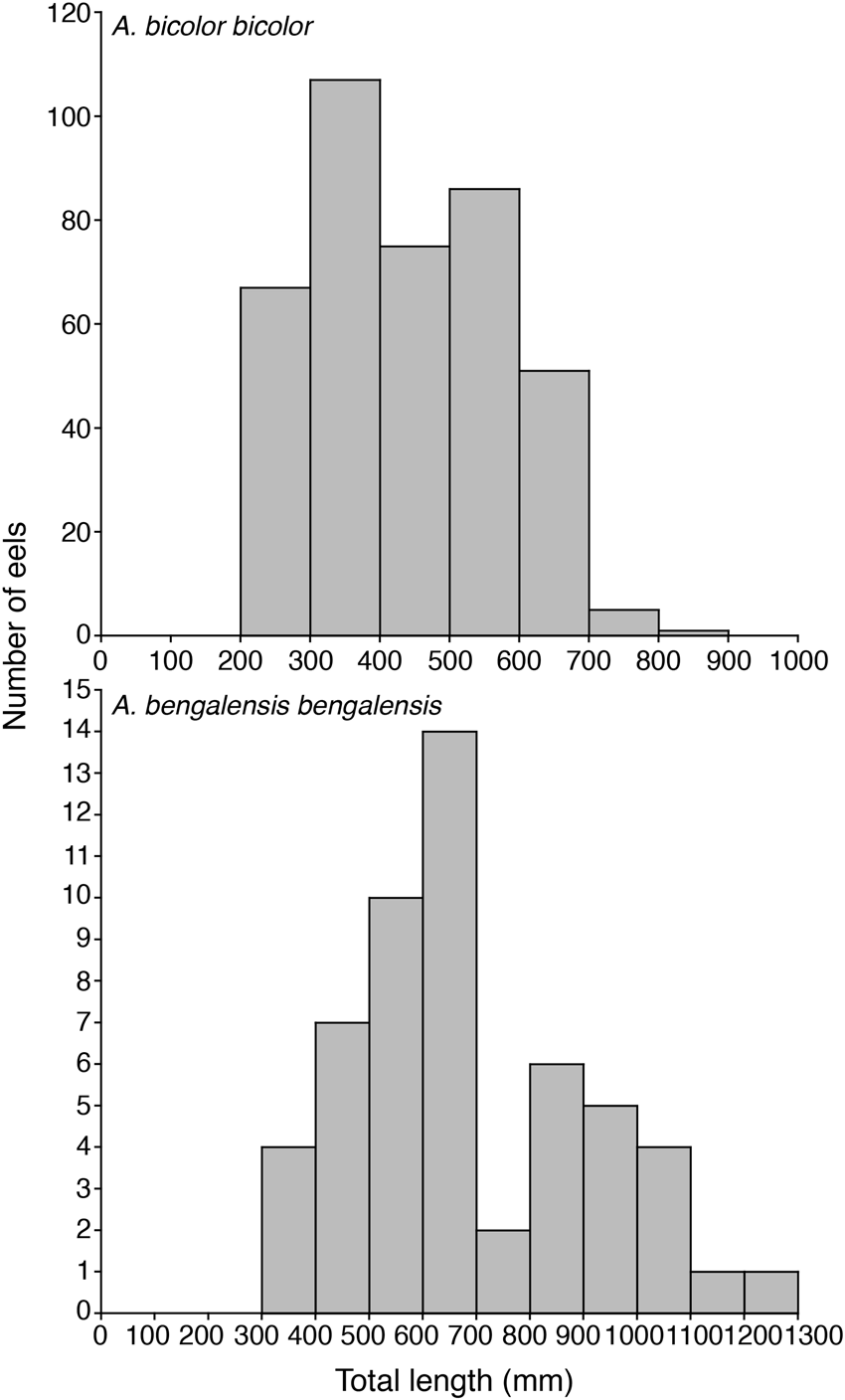
Morphological characteristics. Frequency distribution of total length in all fish for *Anguilla bicolor bicolor* (top) and *A.bengalensis bengalensis* (bottom).

A total of 408, 54 and one specimens were identified belonging to *Anguilla bicolor bicolor*, *A. bengalensis bengalensis* and *A. marmorata*, respectively (Table 1). The overall TL and BW in *A. bicolor bicolor* ranged from 203 mm to 810 mm and from 9.8 g to 1270 g, respectively (Fig. 1, Table 1). Those in *A. bengalensis bengalensis* ranged from 357 mm to 1295 mm and from 51.8 g to 4400 g, respectively (Table 1). TL and BW in one specimen of *A. marmorata* were 904 mm and 2335 g, respectively (Fig. 1, Table 1). Maturation stages of *A. bicolor bicolor* and *A. bengalensis bengalensis* were found to vary widely from Stage I (primary stage) to Stage V (final stage for spawning) (Table 1). These results suggest that the catching methods for anguillid eels in the present study would be suitable for various growth and maturation stages.

**Table 1:** Morphological characteristics of specimens used in the present study.

Species	Location	Sex	Sample size	Total length (mm)		Body weight (g)		Maturation stage range
				mean ± SD	range	mean ± SD	range	
*A. bicolor bicolor*	Temurun, Langkawi Islands, Kedah*	female	1	732		805		II
	Ayer Hitam, Kedah	all	13	638 ± 48.5	568–705	557 ± 145	362–824	
		female	12	639 ± 50.6	568–705	558 ± 152	362–824	II–IV
		unknown	1	626		553		
	Kampung Permatang Bongor, Penang	female	1	625		425		II
	Penang Island, Penang**	all	392	441 ± 133	203–810	195 ± 182	9.8–1270	
	(12 sites)	female	279	492 ± 118	219–810	252 ± 187	17.3–1270	I–V
		male	23	346 ± 71.4	215–558	74.9 ± 37.1	13.6–154	I–III
		unknown	90	290 ± 54.3	203–518	39.7 ± 28.8	9.8–177	
	Hujung Rintis, Perak	female	1	679		571		
	(downstream, estuary, tidal zone)							
*A. bengalensis bengalensis*	Temurun, Langkawi Islands, Kedah*	unknown	2		462–492		155–205	
	Penang Island, Penang**	all	42	594 ± 142	357–920	484 ± 459	51.8–2340	
	(8 sites)	female	23	691 ± 107	487–920	722 ± 500	257–2340	I–V
		male	6	467 ± 66.3	357–557	160 ± 99.2	51.8–313	I–II
		unknown	13	481 ± 80.0	375–610	212 ± 121	65.8–445	
	Kuala Kangsar, Perak	female	10	983 ± 130	830–1295	2110 ± 972	1001–4400	II–V
	(upstream, non tidal effects)							
*A. marmorata*	Penang Island, Penang	female	1	904		2335		IV
	(midstream, non tidal effects)							

*The data was cited from Arai and Wong^29^.

**The details of location in the Penang Island are summarized in Table 2.

### Species distribution in Penang Island and northwestern Peninsular Malaysia

**Figure 2:**
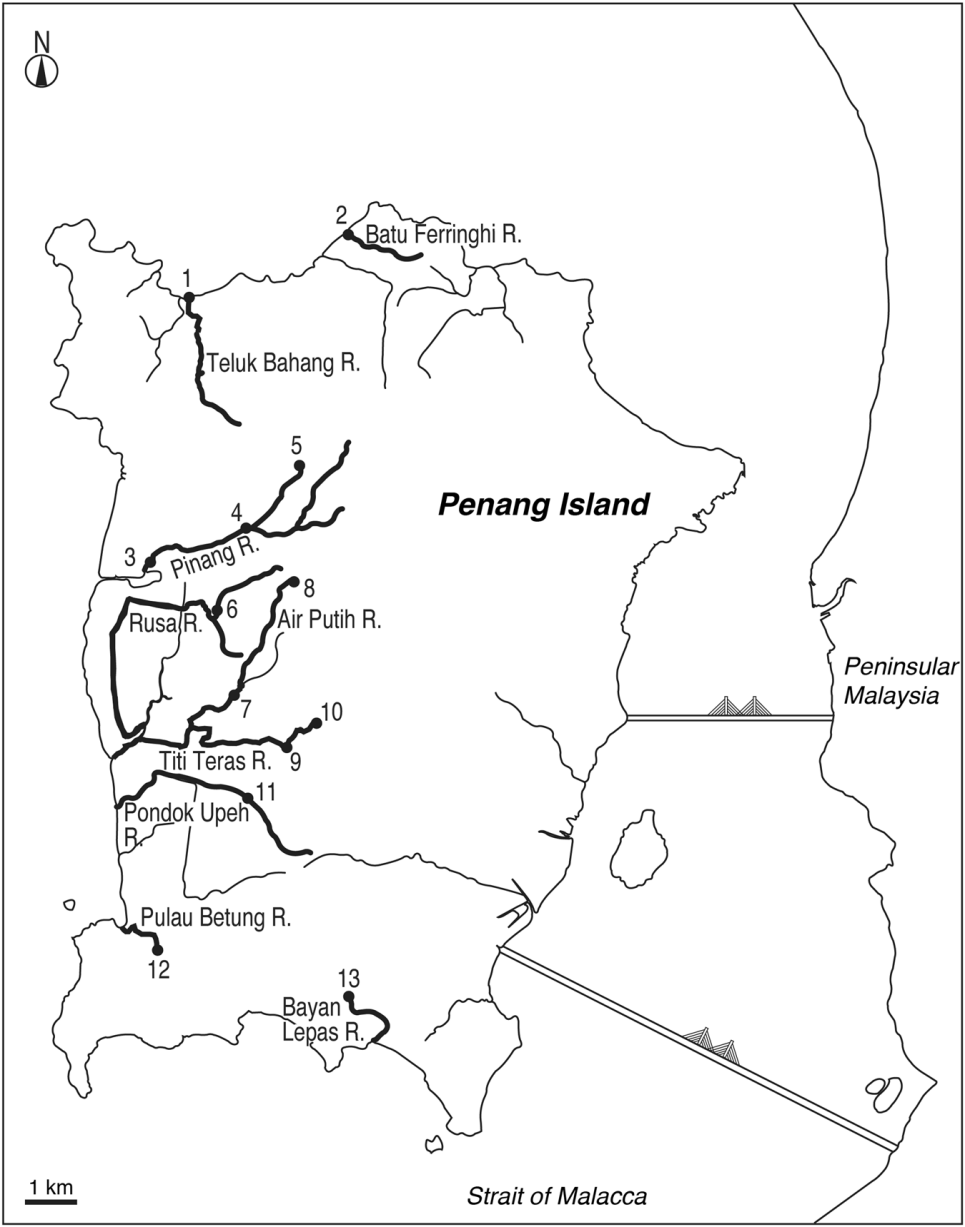
Different habitat use in freshwater ecosystem. Species composition of *Anguilla bicolor bicolor* and *A.bengalensis bengalensis* collected in the 13 sites in the Penang Island, Peninsular Malaysia. 1, Teluk Bahang in the downstream area of the Teluk Bahang River, 2, Batu Ferringhi in the downstream area of the Batu Ferringhi River, 3, Kuala Sungai Pinang in the downstream area of the Pinang River, 4, Kampung Sungai Pinang in the midstream area of the Pinang River, 5, Titi Kerawang Waterfall in the upstream area of the Pinang River, 6, Kampung Sungai Rusa in the midstream area of the Rusa River, 7, Air Putih in the midstream area of the Air Putih River, 8, Bandar Baru Air Putih in the upstream area of the Air Putih River, 9, Titi Teras in the midstream area of the Titi Teras River, 10, Titi Serong in the upstream area of the Titi Teras River, 11, Pondok Upeh in the midstream area of the Pondok Upeh River, 12, Pulau Betung in the midstream area of the Pulau Betung River, 13, Bayan Lepas in the midstream area of the Bayan Lepas River. The map was traced by the author used the Adobe Illustrator CS6 referring Google Maps 2016 (Map data ©2016 Google; https://maps.google.com/).

**Figure 3:**
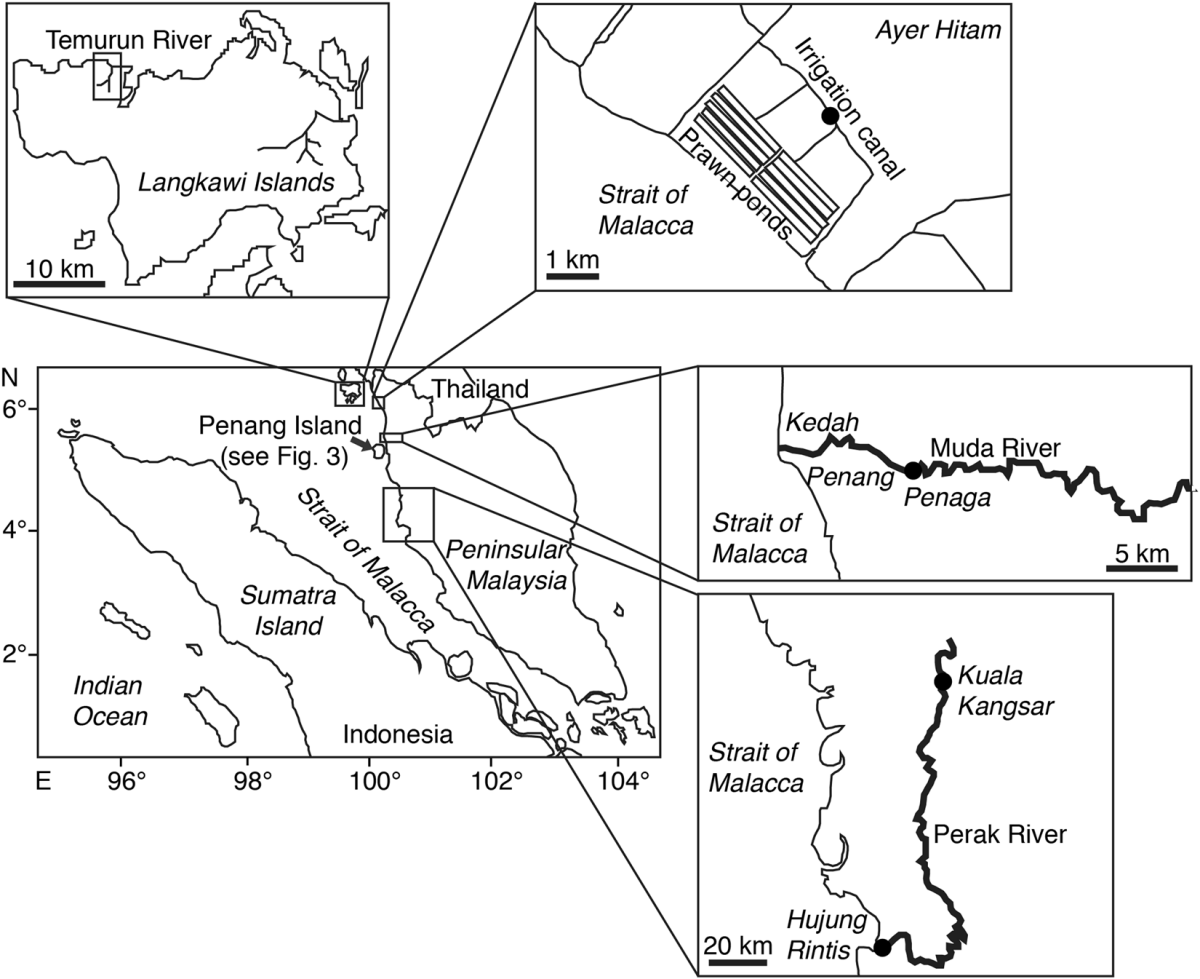
Locations where tropical anguillid eels were collected in the Penang Island. Map showing the collection sites of the tropical anguillid eels in Penang Island in the northwestern Peninsular Malaysia between November 2012 and January 2016. 1, Teluk Bahang in the downstream area of the Teluk Bahang River, 2, Batu Ferringhi in the downstream area of the Batu Ferringhi River, 3, Kuala Sungai Pinang in the downstream area of the Pinang River, 4, Kampung Sungai Pinang in the midstream area of the Pinang River, 5, Titi Kerawang Waterfall in the upstream area of the Pinang River, 6, Kampung Sungai Rusa in the midstream area of the Rusa River, 7, Air Putih in the midstream area of the Air Putih River, 8, Bandar Baru Air Putih in the upstream area of the Air Putih River, 9, Titi Teras in the midstream area of the Titi Teras River, 10, Titi Serong in the upstream area of the Titi Teras River, 11, Pondok Upeh in the midstream area of the Pondok Upeh River, 12, Pulau Betung in the midstream area of the Pulau Betung River, 13, Bayan Lepas in the midstream area of the Bayan Lepas River. The map was traced by the author used the Adobe Illustrator CS6 referring Google Maps 2016 (Map data ©2016 Google; https://maps.google.com/).

*Anguilla bicolor bicolor* was found in 12 out of 13 sites in Penang Island (Figs 2, 3). The species was collected from upstream to downstream of each river and in both tidal and no tidal zones (Tables 2, 3). The occurrence of *A. bengalensis bengalensis* was restricted (8 of 13 sites) from upstream to midstream regions with no tidal zones (Tables 2, 4). Although the habitats of *A. bicolor bicolor* and *A. bengalensis bengalensis* overlapped, *A. bicolor bicolor* was more abundant than *A. bengalensis bengalensis* in many sites (Tables 3, 4, Fig. 3). *A. bengalensis bengalensis* was most abundant in Titi Kerawang Waterfall which is the uppermost part of the Pinang River (5; Table 4, Fig. 3). Such a trend was found in Titi Serong as well which lies upstream of Titi Teras River (10; Table 4, Fig. 3). Furthermore, the midstream of Pinang River where one *A. bengalensis bengalensis* was collected (Fig. 3), have higher elevations than other sites (Table 2). The area of Titi Kerawang Waterfall had the highest elevation ranging 174–217 m; the second highest elevation was in Titi Serong being 50–75 m and the third highest was in the area midstream of Pinang River being 36–38 m (Table 2). Furthermore, temperatures of these sites were lower than that in other sites. The lowest temperature was found in Titi Kerawang Waterfall ranging 23.6 to 24.2 °C and the second was Titi Serong at 25.8 °C in the midstream region of Pinang River (Table 2).

**Table 2:** Sampling location details in Penang Island.

No.	Location	River name	Tidal influence	Elevation (m)	Depth (m)	Salinity (‰)	Water temperature (°C)	Habitat characteristics
1	Teluk Bahang	Teluk Bahang	tidal	2–10	0.1–1.8	27.8	27.5–28.5	moderate flowing water, concrete structure
	(downstream)					(high tide)		lining in the stream banks
	05°27′ 27. N, 100°12′ 30. E					0.04–1.93		
						(low tide)		
2	Batu Ferringhi	Batu Feringhi	tidal	2–8	0.1–0.5	0.06–0.09	26.1–26.7	moderate flowing water, concrete structure
	(downstream)					(low tide)		lining in the stream banks and small drain
	05°28' 00. N, 100°14′ 30. E							
3	Kuala Sungai Pinang	Pinang	tidal	4–11	0.5–1.5	0.25–0.31	26.7–29.9	control by irrigation gate,
	(downstream)					(inside gate)		plantation area along the river
	05°23′ 14. N, 100°11′ 44. E					28.89		
4	Kampung Sungai Pinang	Pinang	no tidal	36–38	0.2–2	0.01	27.0	fast flowing water, bedrock and coarse sand
	(midstream)							in shallow and small stream
	05°24′06. N, 100°13′09. E							
5	Titi Kerawang Waterfall	Pinang	no tidal	174–217	0.2–2.5	0.01	23.6–24.2	fast flowing water, bedrock and coarse sand
	(upstream)							
	05°24′12. N, 100°13′21. E							
6	Kampung Sungai Rusa	Rusa	no tidal	9–13	0.2–0.5	0.02	27.7	moderate flowing water,
	(midstream)							small rocks in the shallow stream
	05°23′10. N, 100°12′45. E							
7	Air Putih	Air Putih	no tidal	14–15	0.5–1.5	0.02	26.3	fast flowing water, small stream with
	(midstream)							bedrock and coarse sand,
	05° 21′ 44 N, 100° 13′ 13. E							concrete structure along the river
8	Bandar Baru Air Putih	Air Putih	no tidal	14–15	0.2–1	0.01	26.8	fast flowing water, small stream with
	(upstream)							bedrock and coarse sand,
	05°22′08. N, 100°13′25. E							concrete structure along the river
9	Titi Teras	Titi Teras	no tidal	19–23	0.5–2	0.02	26.5	moderate flowing water,
	(midstream)							concrete structure along the river
	05°21′37. N, 100° 13′19. E							
10	Titi Serong	Titi Teras	no tidal	50–75	0.2–0.5	0.02	25.8	fast flowing water, bedrock and coarse sand,
	(upstream)							small stream
	05°21′54. N, 100°14′43 E							
11	Pondok Upeh	Pondok Upeh	no tidal	13–15	0.1–0.5	0.03	28	moderate flowing water, mall stream with
	(midstream)							bedrock and coarse sand in shallow water
	05°20′57. N, 100°14′11. E							
12	Pulau Betung	Pulau Betung	tidal	5–10	0.5–1	0.15	30.2	moderate flowing water, mangrove forest
	(midstream)					(low tide)		
	05°21′07. N, 100°12′33. E							
13	Bayan Lepas	Bayan Lepas	no tidal	12–14	0.5–1	0.03	26.9	Moderate flowing water, small stream with
	(midstream)							bedrock and coarse sand in shallow water
	05°19′27. N, 100°16′01. E

**Table 3:** Number of eels in each size group (mm) of *A. bicolor bicolor* collected in each site in Penang Island, Peninsular Malaysia.

No.	Location	Total number	200–299	300–399	400–499	500–599	600–699	700–799	800–899
1	Teluk Bahang	7		2	2	3			
2	Batu Ferringhi	7		2		5			
3	Kuala Sungai Pinang	83	1	20	16	24	18	3	1
4	Kampung Sungai Pinang								
5	Titi Kerawang Waterfall	4		1	2	1			
6	Kampung Sungai Rusa	7			4	2	1		
7	Kampung Titi Teras	20	11	6		2	1		
8	Kampung Pokok Manggis	4				2	2		
9	Balik Pulau	215	55	75	47	24	14		
10	Kampung Titi Serong	2			1	1			
11	Pondok Upeh	4		1	1	2			
12	Pulau Betong	11				6	5		
13	Bayan Lepas	27			2	14	9	2

**Table 4:** Number of eels in each size group (mm) of *A. bengalensis bengalensis* collected in each site in Penang Island, Peninsular Malaysia.

No.	Location	Total number	300–399	400–499	500–599	600–699	700–799	800–899	900–999
1	Teluk Bahang								
2	Batu Ferringhi								
3	Kuala Sungai Pinang								
4	Kampung Sungai Pinang	1			1				
5	Titi Kerawang Waterfall	20	3	1	5	8		2	1
6	Kampung Sungai Rusa	2	1	1					
7	Kampung Titi Teras								
8	Kampung Pokok Manggis	2		1		1			
9	Balik Pulau	2		1		1			
10	Kampung Titi Serong	8		2	3	1	1	1	
11	Pondok Upeh	6		2		2		1	1
12	Pulau Betong								
13	Bayan Lepas	1					1		

**Figure 4:**
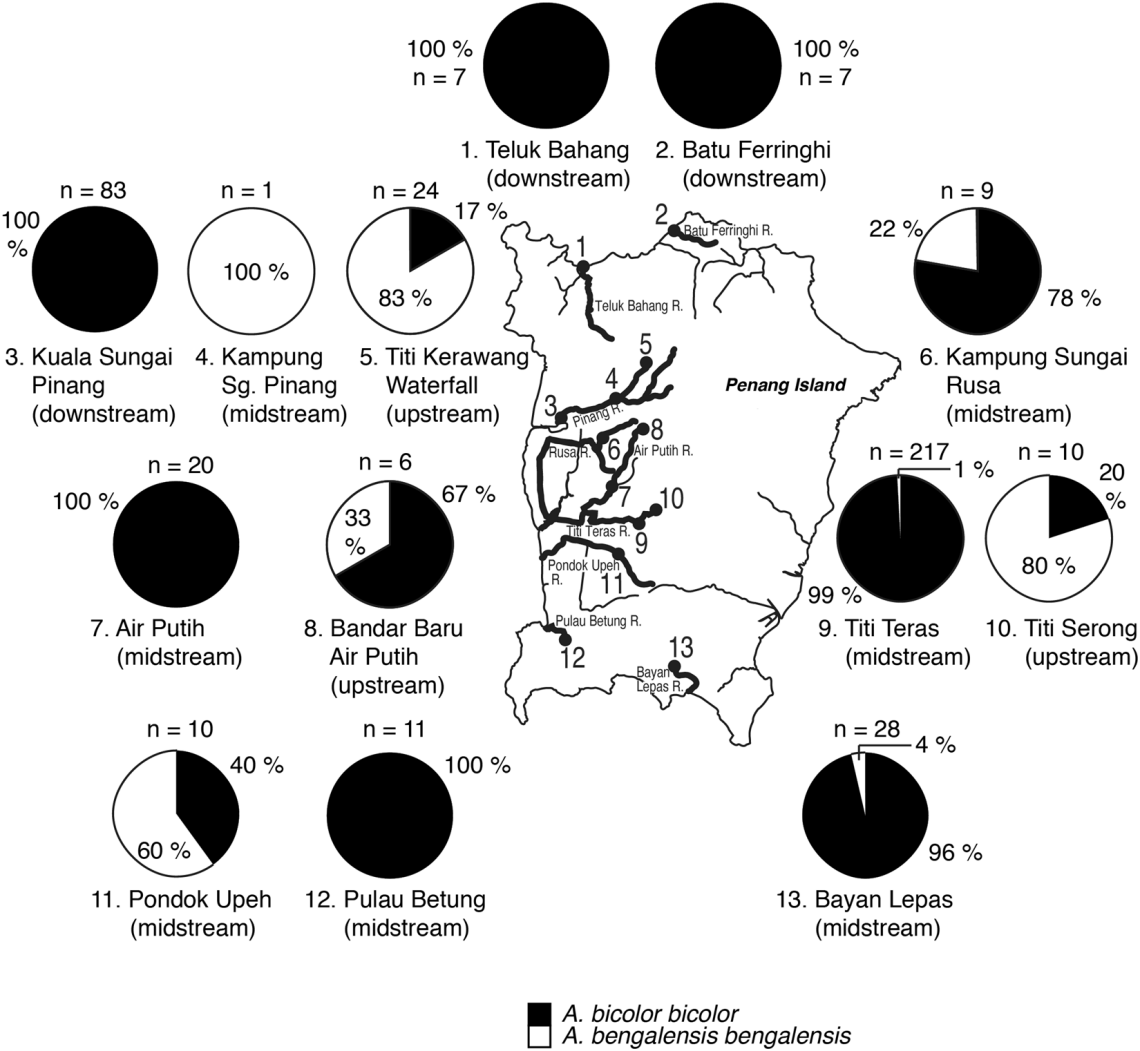
Locations where tropical anguillid eels were collected in the northwestern Peninsular Malaysia. Map showing the collection sites of tropical anguillid eels in northwestern Peninsular Malaysia between November 2012 and January 2016. The map was traced by the author using the Adobe Illustrator CS6 referring Google Maps 2016 (Map data ©2016 Google; https://maps.google.com/).

In Perak River, 10 specimens of *A. bengalensis bengalensis* were collected in Kuala Kangsar which is an upstream area with no tidal zones, but no *A. bicolor bicolor* was collected (Table 1, Fig. 4). Alternatively, one *A. bicolor bicolor* was collected in Hujung Rintis which is the estuarine part, while no *A. bengalensis bengalensis* was collected (Table 1, Fig. 4). In Ayer Hitam and Kampung Permatang Bongor in Penang State, which are downstream and midstream areas, only *A. bicolor bicolor* and no *A. bengalensis bengalensis* was collected (Table 1, Fig. 4).

### Size distribution in Penang Island

In Penang Island, eels of various size groups were collected in each sampling site belonging to both *Anguilla bicolor bicolor* and *A. bengalensis bengalensis* species (Tables 3, 4). In Kuala Sungai Pinang, Bandar Baru Air Putih, Titi Teras and Bayan Lepas, where more than 20 *A. bicolor bicolor* eels were collected, two- (400 mm to 800 mm) to four-fold (200 mm to 800 mm) difference in TL were observed in a same area (Table 3). Such tendency was also observed in Titi Kerawang Waterfall in the case of *A. bengalensis bengalensis* where the difference in TL was approximately three-fold (i.e., 300 mm to 900 mm) (Table 4). The other sites where the sample sizes were less than 20 also had similar tendencies in that the eel sizes were variable and the eels did not fall in the same size group in each location (Tables 3, 4).

## Discussion

This study is the first and the comprehensive description of the species composition, diversity, distribution and habitat use among the anguillid eels in tropical waters, which has accurate and valid species identification. Three species, *Anguilla bicolor bicolor*, *A. bengalensis bengalensis* and *A. marmorata*, were found to occur in the northwestern peninsular area. Overall, the dominant species was *A. bicolor bicolor* constituting 88.1% of the total eels, the second was *A. bengalensis bengalensis* at 11.7%, while *A. marmorata* was the least abundant at 0.2% (Table 1). Comprehensive studies by Ege^1^ have discussed anguillid species diversity, geographic distribution and abundance in the world and have revealed that the highest diversity of Anguillidae occurs around Indonesian and Malaysian waters. However, for Malaysia, there is relatively little information available on the various aspects of eel biology including species composition, distribution, life history and migration. Malaysia should not be excluded as a study area, as it is one of the important geographical niches of Anguillidae. Thus, eel biology research in Malaysia could provide details about their species diversity, evolutionary pathway and life history.

According to previous studies, tropical eel species *Anguilla bicolor bicolor*, *A. bengalensis bengalensis* and *A. marmorata* have been found in Peninsular Malaysia^22–30^. However, these studies by Ng and Ng^22^, Ahmad and Lim^23^, Azmir and Samat^24^ and Hamzah *et al*.^30^ did not include comprehensive identification methods for the anguillid species. In their study, Ahmad and Lim^23^ had indicated that a tropical mottled eel *Anguilla marmorata* had been found in Peninsular Malaysia but after reexamination of a number of key characteristics of the preserved specimen, the eel was identified as *A. bengalensis bengalensis* instead^27^. The occurrence of *A. bengalensis bengalensis* was first recorded in Malaysian waters. Species misidentification in previous studies may have been due to insufficient data from analysis using morphological characteristics. Hence, the identification of eels at the species level using solely visual observation has been known to be difficult because of their similarities and overlapping morphological characteristics, particularly of tropical Anguillidae^1, 2^. In fact, the difficulty in distinguishing *A. marmorata* from *A. bengalensis bengalensis* is augmented by their overlapping morphological characteristics, which cause further identification ambiguities^29^. Indeed, many studies had misidentified *A. marmorata* by using their variegated skin only without sufficient analysis of morphological characteristics^22–24, 30^. Recently, Arai *et al*.^28^ and Arai and Wong^29^ have reported the first occurrence of *A. bengalensis bengalensis* in the Malaysian waters including the Penag and the Langkawi islands and it was confirmed by both morphological and molecular genetic analyses. Furthermore, their studies also highlighted the limitations of tropical eel species identification based solely on morphological analyses. Therefore, the previous reported *A. marmorata* specimens by Ng and Ng^22^, Ahmad and Lim^23^, Azmir and Samat^24^ and Hamzah *et al*.^30^ could have been misidentified, and may have actually been *A. bengalensis bengalensis* specimens. Thus, to validate the identification of the tropical eel species for further biological and ecological studies, it is crucial to utilize both morphological and molecular genetic analyses.

*Anguilla bicolor bicolor* and *A. bengalensis bengalensis* coexisted in most of habitats of Penag Island (Fig. 3), and might share the same niches, use the same demersal habitats and forage for the same prey. However, each species appears to have different habitat preference and/or habitat use. *A. bicolor bicolor* were able to inhabit various environments from upstream to downstream regions of rivers with preference for midstream to downstream areas. However, *A. bengalensis bengalensis* was restricted to midstream to upstream regions without tidal effects and was mainly found in the uppermost streams with higher elevation and cooler water. Such difference in habitat use was found in Perak River, as well, where *A. bicolor bicolor* was in the estuarine part and *A. bengalensis bengalensis* was found in the upstream area only. *A. bicolor bicolor* could also be found only in the downstream areas of Kedah State. These results suggest that habitat use may be a plastic response to local conditions. The area of Penang Island is 293 km^2^ and the rivers are much narrower and shorter than Perak River and Muda River in the Peninsular Malaysia. Therefore, both eel species may need to share their habitat in Penang Island, with less habitat partitioning. Nevertheless, the rivers in the peninsular area might have enough carrying capacity to enable the species to settle into the environmental habitats according to their preferences with regards to factors such as elevation, temperature and salinity. Interspecific competition was thought to play an important role in regulating the habitat use and population size between *A. japonica* and *A. marmorata* in Taiwan^31^. *A. japonica* is dominant in the lower reaches of the river and estuary, while *A. marmorata* dominates in the middle to upper reaches of the river^31^. These mechanisms might exist to avoid interspecific competition and attain maximum benefit for each eel species in the river. However, the distribution and habitat patterns in *A. bicolor bicolor* and *A. bengalensis bengalensis* in Penang Island did not support the interspecific competition hypothesis, but rather suggested that these species might be opportunistic in their habitat use. Recent otolith microchemical studies have found that almost all *A. marmorata* and *A. bicolor pacifica* were primarily estuarine residents occurring sympatrically in a river system in Vietnam^32^. The migratory patterns of *A. bicolor bicolor* were mainly of the switch type involving moving from freshwater to seawater environments in a lagoon in Indonesia^18, 19^. In contrast, the other eels resided consistently in either seawater or brackish water. No *A. bicolor bicolor* showed constant freshwater residence^18, 19^ (Table 5). Contrastingly Chino and Arai^18, 19^, approximately 40% *A. bicolor pacifica* in the Philippines showed freshwater residence, while most showed estuarine residence (60%)^33^. *A. bicolor bicolor* collected in the upper reach of complete freshwater area in Bukit Merah in Krau River of Peninsular Malaysia, was reported to have a fully freshwater life history^25^ (Table 5). Thus, the habitat preference might be different between sites due to inter-species interactions and intra-specific plasticity to local environmental conditions (Table 5). These results suggest that habitat use in the tropical anguillid eels might be more influenced by such ambient environmental factors as salinity, temperature, elevation, river size and carrying capacity than ecological competition, such as interspecific competition. Further studies should be undertaken to examine the environmental factors at each site with detailed distribution and migratory history analyses along the river system to elucidate the valid mechanisms of habitat use in each species.

**Table 5:** Migratory history of *Anguilla bicolor bicolor* and *A. bicolor pacifica*.

Species	Country	Migratory types	Reference
A. bicolor bicolor*	Philippines	freshwater/estuarine	33
*A. bicolor pacifica*	Philippines	estuarine/marine	33
*A. bicolor bicolor*	Indonesia	estuarine/marine	18
*A. bicolor bicolor*	Indonesia	estuarine/marine	19
*A. bicolor pacifica*	Vietnam	estuarine/marine	32
*A. bicolor bicolor*	Malaysia	freshwater	25

*A. bicolor bicolor could be *A. bicolor pacifica* as suggested in Arai and Chino^7^.

In each habitat, there was not only the coexistence of two species but also specimens of various sizes were found within the same species (Tables 3, 4). The present study suggests that minimal intraspecies competition among various growth stages might have occurred in their habitats. However, different habitat uses along the eel growth were found among temperate eels^34–40^. Such habitat preferences during the eel growth was also reported in *Anguilla anguilla* ^34^. The eels of sizes less than 160 mm had a ubiquitous behaviour. The eels of intermediate sizes between 160 mm and 360 mm showed a progressive change of habitat preference. These eels preferred deeper habitats while the large eels, more than 360 mm, had a strong preference for large ditches with deep water. The general pattern is for *A. anguilla* to shift progressively to deeper habitats as they grew. These results are also consistent with other studies that have found habitat shifts along the eel growth. The smaller juvenile eels of *A. australis* appeared to be more abundant closer to shore, but the larger eels were more evenly distributed in a lake where they may be able to use a wider range of substrate types^35^. Similarly, young eels of *A. australis* were much more abundant in the lake margins than in the open water where the larger eels predominated^36^. This type of habitat shift may be related to feeding, and *A. australis* yellow eels were found to shift to being almost exclusively piscivorous at sizes greater than 500 mm^37^. Eels of this size would be predominantly females. The pattern is also similar in the case *A. rostrata*, which become piscivorous at a size that only female eels achieve^38^. Although all eels are feeding generalists^39^, smaller eels are more restricted because of their inability to ingest larger food items such as fish. Eels do not usually undergo a dramatic niche shift, but as their size increases, so does their niche breadth as a result of the inclusion of piscivory^40^. Although this would be similar in *A. bicolor bicolor* and *A. bengalensis bengalensis*, this study did not find habitat shifts based on their life stages. On the other hand, temperate eels typically have quite localized home ranges with some movement to different areas. For example, studies of New Zealand eels reported that the large eels have relatively small home ranges and typically only move short distances within the streams^41–43^. Therefore, smaller eels might freely use a habitat that the bigger eels had stayed in. These results suggest that tropical eels might be able to stay in various habitats, not in an obligatory manner but facultatively during their growth and migration.

In this study, we could only observe anguillid eels in the northwestern parts of the Peninsular Malaysia. However, no eels could be observed from the southwestern to the eastern peninsular in this four year research. This pattern of geographical distribution and species composition of the anguillid eels might be due to the transportation mechanisms from spawning areas to their fresh water growth habitats in the peninsular. Recently, we discovered *A. bicolor bicolor* in the western and northern parts of the peninsular^25^. According to Jespersen^44^, the occurrence of preleptocephalus to metamorphosing stages in anguillid eels in waters off Sumatra supports the populations of the eels that are distributed in Java and Sumatra, and they may have their spawning area situated off the south-western coast of Sumatra, close to the distribution area of the freshwater stage (Fig. 1). Jespersen^44^ reported many leptocephali in the waters of west Sumatra, and 85% of the individuals ranging from 20 to 56 mm could be distinguished as the short-finned type, which were probably *A. bicolor bicolor*. The other 15% long-finned type would be *A. bengalensis bengalensis* and *A. marmorata* ^1^. The composition found near Sumatra was similar to that in northwestern Malaysia in the present study. Thus, *A. bicolor bicolor*, *A. bengalensis bengalensis* and *A. marmorata* in the northwestern coast of Malaysia might have originated from spawning areas from near Sumatra or the same populations. However, the distance between the spawning area and recruitment area in the southwestern and the eastern Peninsular Malaysia is considerably longer than the distance between the islands of Java and Sumatra, and there are no oceanic current transporting larvae to the eastern coast from of Sumatra^45^. Thus, no distribution of the anguillid eels in the eastern Sumatra supports this speculation. Further research on the spawning grounds, larval transportation and recruitment in this region are needed to gain a better understanding of the geographic distribution and ecology of the anguillid tropical eels in the eastern Indian Ocean.

## Methods

### Eel collection in Peninsular Malaysia

We collected the eels in Peninsula Malaysia between November 2012 and January 2016. This qualitative study was conducted mainly through interviews with local fishermen, buyers and officials and *in situ* samplings with local fishermen for four years. A total of 463 specimens were collected by local fishermen mainly in Penang Island (435 specimens) of the northwestern Peninsular Malaysia (Table 1, Figs 2, 4). No eels were collected from the mid-southwestern and the eastern parts of the peninsular (Fig. 4). The eels were collected by angling and with eel traps at night. All specimens were fixed immediately after collecting by freezing. The water temperature and salinity were measured sporadically at each sampling site (Table 2). No specific permission was required for these locations/activities, as the eel species involved are not endangered or protected and because the collection areas did not require any permits to collect these animals. Our protocol was in accordance with the guide for animal experimentation at Universiti Malaysia Terengganu (UMT) and fish-handling approval was granted by the animal experiment committee of UMT. Furthermore, the UMT Ethics Committee also approved all experimental protocols in this study.

### Eels in Penang Island

In Penang Island, a total of 435 specimens were collected from nine rivers at thirteen sites (Figs 2, 3). Each sampling site was divided into upstream (near headwater areas without tidal influence), midstream (intermediate area of river) and downstream (less than 0.2 km from river mouth with tidal influence). These rivers are Batu Ferringhi River, Teluk Bahang River, Pinang River, Rusa River, Air Putih River, Titi Teras River, Pondok Upeh River, Pulau Betung River, and Bayan Lepas River (Fig. 2, Table 2). The four sites near the Pinang River areas were located downstream (3. Kuala Sungai Pinang), midstream (4. Kampung Sungai Pinang) and upstream (5. Titi Kerawang Waterfall) of Pinang and Rusa rivers (midstream) (Fig. 2). The four sites near Air Putih and Titi Teras rives areas include Bandar Baru Air Putih (8) (upstream) and Air Putih (7) (midstream) and Titi Serong (10) (upstream) and Titi Teras (9) (midstream) (Fig. 2). Pondok Upeh (11) is located on the midstream in the Pondok Upeh River. Sampling sites of Batu Ferringhi (2) and Teluk Bahang (1) are located in the downstream areas of each river while those of Pulau Betung (12) and Bayan Lepas (13) are located in the midstream areas (Fig. 2, Table 2). A total of 4 locations-Batu Ferringhi, Teluk Bahang, Kuala Sg. Pinang and Pulau Betung-were influenced by rising tide, while of 9 locations-TitiKerawang Waterfall, Kampung Sungai Pinang, Kampung Sungai Rusa, Bandar Baru Air Putih, Air Putih, Titi Serong, Titi Teras, Pondok Upeh and Bayan Lepas-were not influenced by the tide (Table 2).

### Eels from other sites in northwestern Peninsular Malaysia

In addition to the eels collected in Penang Island, 28 of the 463 specimens were collected in Langkawi Islands (3 specimens; Arai and Wong^29^), Ayer Hitam (13 specimens) and Penaga (1 specimen) in Kedah State and Hujung Rintis (1 specimen) and Kuala Kangsar (10 specimens) in Perak State (Table 1, Fig. 4).

Eels of Ayer Hitam were collected in the downstream area of irrigation canals (3.7–4.8 km from estuary) where the rising tide could be influenced and controlled by the irrigation gates. The depth and elevation ranged from 0.5 m to 1.5 m and from 3 m to 5 m, respectively. One eel from Kampung Permatang Bongor was collected midstream of Muda River with no influence of the rising tide (12 km from estuary). The depth and elevation ranged from 1.9 m to 2.2 m and from 6 m to 8 m, respectively.

Perak River is the second largest river in Peninsular Malaysia with a total length of approximately 400 km. One eel from Hujung Rintis was collected downstream of Perak River (7.5 km from the river mouth) where it was influenced by the rising tide. The salinity and water temperature at the low tide and high tide ranged from 0.05‰ to 0.75‰ and from 29.9 °C to 30.5 °C, respectively. The water level varied a lot between low tide (8–9 m) and high tide (16–18 m). The elevation ranged from 0 m to 3 m. Eels from Kuala Kangsar were collected upstream of Perak River (170–176 km from the river mouth) with no influence from the rising tide, and with 0.01‰ to 0.05‰ salinity. The water temperature ranged from 28.1 °C to 36.3 °C. The depth and elevation ranged from 1 m to 13 m and from 37 m to 52 m, respectively.

### Sample preparation and species identification

After the eels were collected, biological parameters, such as total length (TL) and body weight (BW), were measured (Table 1). The sex of each eel was determined by visual and histological observations of the gonads. The sex and maturation stage of all eels in Penang Island were referred from Arai and Abdul Kadir^20^. Maturation stages correspond to a growth phase (stages I and II), a pre-migrant phase (III) and two migrating phases (IV and V) in female. In male, maturation stages correspond to immature (stage I), early maturation (stage II) and mid-maturation (stage III) stages.

Because of the difficulty to accurately identify tropical eels solely based on morphological analyses, Arai^2^ suggested that accurate tropical eel species identification needed to be validate by molecular genetic analysis after morphological observation. Therefore, the anguillid eels found in the northwestern Peninsular Malaysia were first identified based on morphological analysis, and their identities were further validated by analysing the mitochondrial cytochrome oxidase subunit I (COI) sequence and 16 S ribosomal RNA (16 S rRNA), according to Arai *et al*.^28^ and Arai and Wong^29^.
